# Can Religiosity and Social Support Explain Effects of Trait Emotional Intelligence on Health-Related Quality of Life: A Cross-Cultural Study

**DOI:** 10.1007/s10943-020-01163-9

**Published:** 2021-01-07

**Authors:** Hina Ghafoor, Peter Nordbeck, Oliver Ritter, Paul Pauli, Stefan M. Schulz

**Affiliations:** 1 Department of Psychology (Biological Psychology, Clinical Psychology, and Psychotherapy), University of Würzburg, Würzburg, Germany; 2 Clinical Psychology, Psychotherapy, and Experimental Psychopathology, Johannes Gutenberg University Mainz, Mainz, Germany; 3 Comprehensive Heart Failure Center, Würzburg, University Hospital of Würzburg, Würzburg, Germany; 4 Klinikum Brandenburg, Department of Cardiology, Nephrology and Pulmology, Medical School Brandenburg, Brandenburg, Germany; 5 Center of Mental Health, University of Würzburg, Würzburg, Germany; 6 Department of Applied Psychology, Riphah International University, Islamabad, Pakistan

**Keywords:** Cross-cultural comparison, Chronic heart failure, Religion, Social support, Trait emotional intelligence, Health-related quality of life

## Abstract

Religion and social support along with trait emotional intelligence (EI) help individuals to reduce stress caused by difficult situations. Their implications may vary across cultures in reference to predicting health-related quality of life (HRQoL). A convenience sample of *N* = 200 chronic heart failure (CHF) patients was recruited at cardiology centers in Germany (*n* = 100) and Pakistan (*n* = 100). Results indicated that trait-EI predicted better mental component of HRQoL in Pakistani and German CHF patients. Friends as social support appeared relevant for German patients only. Qualitative data indicate an internal locus of control in German as compared to Pakistani patients. Strengthening the beneficial role of social support in Pakistani patients is one example of how the current findings may inspire culture-specific treatment to empower patients dealing with the detrimental effects of CHF.

## Introduction

Germany and Pakistan are two countries with distinct cultural differences. German society reinforces an autonomous and independent self as compared to Pakistani culture where an individual grows with a relational and interdependent self. Among other important aspects of culture, the two countries predominantly differ in terms of both religion and social support system.

In Pakistan, religion has influenced every dimension of living from birth until death such as *Azaan* (i.e. call to prayer) or arranging religious sermons. In addition, *Piri*-*mureedi* is the most prevalent aspect that influences Pakistani Muslim society even before its creation. *Pir* is the title given to the hereditary descendants of the Islamic saint whereas *mureed* are their followers. Those who have a belief on *Piri*-*mureedi* follow them in every aspect of life even for the treatment of illnesses particularly in Multan, Pakistan. Multan, where this study recruited Pakistani patients, is famous as city of saints because there are more than 20 shrines of Sufis, which have been taken care by the *Gaddinashine/Pirs*. A large population residing in Multan and further followers from across the country visit the city to celebrate annual ceremonies of these saints as well as to consult the native healers.

In Germany, religion differs considerably between regions. The Politbarometer survey 2016 (see Forschungsgruppe Wahlen, Manheim 2017) reported 68.1% of Catholics vs 22.3% Protestants and 8.2% non-religious in Saarland (Western Germany); 61.5% Protestants vs 3.2% Catholics and 31.2% non-religious in Schleswig–Holstein (Northern Germany); 74.7% non-religious population versus 5.1% Catholics and 18.8% Protestants in Saxony-Anhalt (Eastern Germany). Würzburg, where we recruited most German patients, is famous for the presence of approximately 70 churches and hence named ‘the city of churches’. Yet, legally and practically, Germany became a highly secular state after World War II. Whether one is Catholic, Protestant or atheist is a private matter in Germany, whereas in Pakistan religious belongingness is discussed and practiced in public.

Pargament, Koenig and Perez (2000) have explained the explicit role of religion in coping with stress and have identified two main types of religious coping, namely positive and negative ranging from active religious participation and spiritual connection with the supreme power to interpersonal religious discontent and the vision of a punishing God. In a meta-analysis, Ano and Vasconcelles (2005) have established that individuals (European-Americans, native Americans, Middle Eastern, Asian etc.) who used positive religious coping accomplished more stress-related growth and experienced less depression and anxiety. Similarly, both intrinsic (personal relationship with God) and extrinsic (performing religious rituals and relationships with others) aspects of religion have been found to be strongly associated with mental health and well-being in Pakistani sample (Khan and Watson 2006). In addition, religiosity was significantly positively associated with higher self-esteem among Pakistani OCD patients, highlighting the positive effect of religion (Ghafoor and Mohsin 2013).

Büssing et al. (2009) searched for religious and spiritual coping in secular societies (such as Germany) and explained that concrete engagements in religious activities (conventional practices), developing personal insight (existentialistic practice), meditation (spiritual mind–body practice), and caring for others (humanistic practices) are fundamental components of religious paradigm. These components are reflections of ones’ *locus of control*. For instance, external locus of control in religion is to develop a sense of more control over ones’ lives by trusting the most powerful, loving and responsive God (Krause 2002). In contrast, an internal locus of control is characterized by a conscious and healthy way of living and positive attitudes as evident from existentialistic and humanistic practices (Büssing et al. 2009). Hence, religion and its associated beliefs influence overall living quality of an individual by enabling individuals to perform their assigned duties. However, there are also studies, which claim that religion is not always a supportive coping strategy (Chapman and Steger 2010; Ghafoor et al. 2018). This suggests that not all who suffer from difficult life events find peace in religion. On the one hand, these mixed results may in part be explained by differences in the operationalization of constructs. On the other hand, differences in the importance given to religion in one’s life and the evaluation of oneself as a religious person may help to further understand these variations. In particular, the discrepancy of believing something and the failure to follow it through in practical implementation (e.g., because one’s disease is a hindrance) might influence health-related quality of life (HRQoL) of individuals considerably.

Pakistan and Germany also differ considerably in the role of social relationships, which may provide coping resources in the form of social support. Social relationships (family, friends and significant others) have vital influence on health and well-being across the life span (Antonucci et al. 2013). Living in extended family households (joined family system) is considered a powerful support group and may play a significant role in health care (Itrat et al. 2007). However, nowadays this system has been transforming and shifting to the nuclear family system (Itrat et al. 2007). Perhaps receiving support would be helpful if it fulfills one’s basic needs according to self-determination theory (Ryan and Deci 2000) and stress buffering hypothesis. While extended family households are common in Pakistan, Germans mostly maintain a nuclear family living style. The healthy living style adapted by the family helps to remain organized and stable in times of crisis (Martinez-Montilla et al. 2017) or for example when confronted with a chronic illness such as chronic heart failure (CHF).

However, cultural and environmental factors including religion and social support may only support well-being when an individual is capable of tapping into these resources appropriately. As shown above, religiosity is not a supportive resource for everyone (e.g., Ghafoor et al. 2018). One of the best studied and most influential personal factors contributing to overall mental and physical health—presumably via the competent recruitment of coping resources—is trait emotional intelligence (EI).

Trait-EI has been defined as constellation of self-perceptions that are located at the lower levels of personality hierarchies (Petrides 2009). There is an extensive literature supporting the fact that trait-EI plays a significant role in ameliorating the negative effects of physical and psychological stressors in all domains of life (Martins et al. 2010; Smith et al. 2012). Notably, self-reported EI was positively associated with increased religious practice (Paek 2006) and intrinsic religiosity (Butt 2014). Religious beliefs have been shown to be a source of better social functioning by influencing the well-being, and various processes of emotion regulation due to their involvement in self-control and increased emotional awareness (McCullough and Willoughby 2009). Trait-EI has been shown to be also positively associated with social capital which is the use of resources and support by means of the relationship with significant others in a particular context (Bozionelos and Bozionelos 2018). These findings indicate that positive effects of high trait-EI on well-being may be explained at least partially by religious affiliation and social support.

CHF has been called a global pandemic (Savarese and Lund 2017). It is characterized by inadequate blood supply due to weakening of heart muscles (American Heart Association 2017) and contributes to high mortality and a relative increase in disease burden all over the world (Ambrosy et al. 2014). The prevalence rate for CHF in Germany has been reported to be 3.96% (Störk et al. 2017). For Pakistan, no exact prevalence rates exist. However, based on data from the Hayatabad Medical Complex Peshawar Pakistan, the percentage of CHF patients can be estimated to be 15% and 23% of all cardiac patients in 2008 and 2010, respectively (Noor et al. 2012). CHF may be accompanied by risk factors such as hypertension, diabetes, cardiomyopathy, depression and anxiety (Chapa et al. 2014). These associated risk factors may disrupt the HRQoL of an individual in addition to the challenge imposed by CHF per se (Zambroski et al. 2005).

The research reviewed above suggests that a person may find solace in religion both in Western and non-Western societies. Moreover, trait-EI might help individuals to cope with the challenges associated with CHF. Specifically, trait-EI may help to recruit support by family or friends, who may serve as a source for increased life satisfaction and happiness (Nguyen et al. 2015).

The present study is aimed at examining these relationships, specifically, the relationship of religiosity, social support and HRQoL with trait-EI, in CHF patients in Germany and Pakistan. Furthermore, it was examined whether trait-EI, discrepancy between religious belief and practice, as well as social support predicted HRQoL. Moreover, considering the differences of both societies, we added qualitative exploration of the patients’ insight into mechanisms underlying CHF as well as traditional/typical ways to cope with their illness in both societies.

## Methods

The study was a cross-sectional survey conducted in Germany and Pakistan. *N* = 200 (*n* = 100 from each country) CHF patients aged 18 years and above, and/or no current or prior diagnosis of psychosis and substance abuse were recruited from the cardiology centers in Pakistan and Germany. Participants were referred to the experimenter by the consultant cardiologist on duty. All participants provided signed-informed consent prior to study participation. The study protocol was approved by local ethics commission, complied with the regulations of the Declaration of Helsinki, and adhered to guidelines for good clinical practice.

In addition to a self-report questionnaire for assessing socio-demographic data, the following set of measures was applied:

1. The *Trait Emotional Intelligence Questionnaire*-*Short Form* (TEIQue-SF, Petrides 2009) was used to measure trait-EI. It is the shorter version of TEIQue, which has 150 items and 15 facets. The TEIQue-SF is comprised of 30 items, representing 2 items from each facet calculating a global trait-EI score as well as four factor scores of well-being, self-control, emotionality and sociability. Scores ranged from 1 (low trait EI) to 7 (high trait EI). In the present study, Cronbach’s Alpha was *α* = .88 for both the Pakistani and the German sample.

2. The *36*-*item Short Form Health Survey Questionnaire* (SF-36, Ware et al. 1993) is a 36 items questionnaire aiming at assessing eight physical and mental dimensions of HRQoL. Scores ranged from *0* (maximal impairment) to *100* (no impairment) and were summarized into a mental and physical summary score. Cronbach’s alpha in the current sample was excellent (*α* = .83 for Pakistan, *α* = .96 for Germany).

Additionally, general information on religion and social support was gathered using four single-item questions in the national languages of Germany and Pakistan, i.e., German and Urdu, respectively, to be answered on 10-point Likert scales. Higher values indicate higher levels of general religiosity and social support. From two questions on religion (‘importance of religion in one’s life’, and ‘personal evaluation of religious affiliation/practices via the question *how religious would you rate yourself*’), a discrepancy score (DS) was calculated by subtracting the self-rating of how important religion is for one’s life minus the self-rating of oneself as a religious practitioner. Higher scores indicate that a person falls short of their ideal when it comes to actual religious practice. Similarly, from two questions on the importance of family and friends in coping (e.g., *how important is/are your family/friends in providing support during stress*), an index of quality of social support (QSS) was obtained by adding them. Higher scores indicate better social support.

Finally, (instead of later), qualitative information was obtained via four open questions concerning illness knowledge, culture-specific methods of treating illness, frequent worries and culture-specific preferred methods of dealing with worries.

## Data Analysis

The analyses were conducted with IBM SPSS Statistics 23 (IBM Corp 2015). Means, standard deviations, and independent sample t-tests were used for continuous variables (e.g., age). Frequencies, percentages, and Chi^2^ tests were carried out for categorical variables. Correlation analysis was carried out to examine the relationship of all variables with trait-EI. Later, hierarchical regression analysis was conducted to check the predictive value of trait-EI, DS, QSS, interaction of trait EI and DS as well as trait EI and QSS for predicting mental and physical component summaries of HRQoL.

For the qualitative responses to the four open questions, two reviewers recoded the content into major uniform semantic categories. Then, Chi^2^ tests were used to identify differences between Germany and Pakistan. Next, to identify differences between individual categories in the two countries, post hoc analysis was carried out. For this purpose, adjusted residuals for each category were transformed into *p*-values and the *p* value was adjusted by dividing .05 by the number of rows x columns for each theme (Grande 2016).

## Results

### Sample

The sample comprised of 100 Pakistani (*n* = 36 female [36%]; Age: *M *= 53.79 years *SD *= 13.77) and 100 German (*n* = 26 female [26%]; Age: *M *= 65.98 years *SD *= 12.20) CHF outpatients. The two samples differ significantly in terms of age: (*t *= − 6.57, *p* ≤ .001) and living arrangements: *Chi*^*2*^(12.85)*, df *= 1*, ϕ* = − .27*, p *< .001. Most of the patients in Pakistan reported living in extended family households (Pakistan: *n* = 49, 49% vs. Germany: *n* = 19, 19%) as opposed to the nuclear family system, which is prevalent in Germany (Germany: *n* = 62, 62% vs. Pakistan: *n* = 50, 50%; missing data: Germany *n* = 19, 19% vs. Pakistan: *n* = 1, 1%). Both samples differ partially significantly (*Chi*^2^ (131.09)*, df *= 38*, ϕ* = .82*, p *< .001) regarding the member providing financial support to the family when controlling for marital status. The results were significant for married (*Chi*^2^ (109.42)*, df *= 27*, ϕ* = .87*, p *< .001) and widowed (*Chi*^2^ (25.73)*, df *= 11, *ϕ* = .91*, p *< .01) category and remained non-significant for single, separated, and divorced individuals. It shows that in the Pakistani sample among married patients, mostly the patient themselves (*n* = 44, 56% self, and *n* = 19, 24% sons) compared to both spousal partners in the German sample (*n* = 21, 32% self and *n* = 40, 62% both spousal partner), whereas among widowers mostly the children (sons; *n* = 17, 89%) in the Pakistani sample compared to patient themselves in the German sample (*n* = 6, 50%) were the members who provide financial support to the family.

Similarly, significant differences were observed regarding the individual who made important family decisions (*Chi*^2^ (87.98), *df* = 39, *ϕ* = .67, *p* < .001). The results were significant for married (*Chi*^2^ (60.76)*, df *= 28*, ϕ* = .65*, p *< .001) and widowed (*Chi*^2^ (21.45)*, df *= 10*, ϕ* = .83*, p *< .05) category and remained non-significant for single, separated, and divorced individuals. It shows that in the Pakistani sample among married patients, mostly the patient themselves (*n* = 49, 63%) compared to both spousal partners in the German sample (*n* = 18, 28%); whereas among widowers mostly the patient themselves (Pakistani: *n* = 11, 59% vs. German: *n* = 4, 33%) were the members who made important family decisions.

All psychometric data were normally distributed as evident from the values of skewness and kurtosis within an acceptable range (between − 2 and + 2; Field 2013; George and Mallery 2010). According to Littles’s test (1988), missing values were missing completely at random (Chi^2^ (248) = 274.38, *p *= 1.20) and accounted for only 1.85% of the data. Missing values were imputed with the 'series means methods’ (Little and Rubin 2002).

### Cultural Differences Regarding the Importance of Religion, Social Support, and Psychological Variables

Table 1 indicates that the Pakistani CHF patients rated themselves as more religious and religiosity as significantly more important as reflected in the IOR score (importance of religion in one’s life). In addition, the DS was significantly higher in the Pakistani than in the German sample indicating discrepancy between religious beliefs and practices. Moreover, the German sample considered friends as a source of support, gave quality of social support (QSS) significantly higher ratings, and were characterized by better physical and mental component summaries of health-related quality of life (HRQoL) as assessed by the SF-36 (Ware et al. 1993).

**Table 1:** Means (M) and standard deviations (SD) of psychological variables for the two cultures (Pakistan vs. Germany) as well as results of group comparisons

	Pakistan (*n* = 100)	Germany (*n* = 100)	*t*	*p*	*Cohen’s d*
	*M (SD, R)*	*M(SD, R)*			
IOR	2.61 (2.12, 9)	− 2.55 (3.60, 10)	12.34	.001***	1.75
Religiosity	1.36 (3.17, 10)	− 1.30 (3.36, 10)	5.76	.001***	.81
Family	.01 (3.30, 10)	.02 (2.85, 10)	− .02	.98	.003
Friends	− .85 (3.64, 10)	.88 (3.01, 10)	− 3.67	.001***	.52
DS	1.26 (4.06, 18)	− 1.25 (1.56, 10)	5.77	.001***	.82
QSS	− .84 (5.40, 20)	.89 (5.07, 20)	− 2.34	.02*	.33
TEIQue-SF	4.69 (.92, 4)	4.71 (.87, 5)	− .12	.90	.02
SF-36 (PCS)	30.85 (13.44, 64)	49.20 (26.64, 90)	− 6.15	.001***	.87
SF-36 (MCS)	47.15 (14.94, 64)	58.66 (21.39, 91)	− 4.41	.001***	.62

*DS* discrepancy score in religious beliefs and practices, *IOR* importance of religiosity, *MCS* mental component summary, *PCS* positive component summary, *QSS* quality of social support, *SF-36* the 36-item Short Form Health Survey Questionnaire, *TEIQue-SF* trait emotional intelligence questionnaire-short form

*p ≤ .05; ***p ≤ .001

Table 2 shows significant differences of correlations of trait-EI with other psychological and cultural variables between the two cultures. In the Pakistani CHF patients, significant positive correlation existed only between trait-EI and importance given to religiosity (IOR) indicating that higher trait-EI was linked to IOR among the Pakistani sample only.

**Table 2:** Correlations of psychological variables and Trait-EI for Pakistani and German CHF patients, and results of Fisher’s z-tests, comparing correlations between the two samples

	IOR.	Religiosity.	Family.	Friends.	DS.	QSS.	MCS.	PCS.
*TEIQue*-*SF*								
Pakistani	.24*	.09	.14	.11	.06	.15	.14	.39**
Germany	− .01	.009	− .02	− .01	− .05	− .02	.31**	.46**
*p*	.03*	.28	.13	.20	.22	.12	.10	.27

*DS* discrepancy score in religious beliefs and practices, *IOR* importance of religiosity, *MCS* mental component summary, *PCS* physical component summary, *QSS* quality of social support, *TEIQue-SF* trait emotional intelligence questionnaire-short form

**p ≤ .01

### Regression Analyses

Hierarchical regression analysis was carried out by forming two blocks. In the first block, age was entered as statistically controlled variable whereas in the second block all predictor variables (trait EI, DS, QSS) along with interaction terms (Trait EI x DS & Trait EI x QSS) were added.

The analysis revealed that for Pakistani sample, the variable age contributed 0% (*R*^2^ = .00) for PCS. However, when statistically controlled for age, the other predictor variables contributed 8.9% change in *R*^2^ (*R*^2^ = .89). The model for Pakistani sample was, however, not significant (*F* (6, 98) = 1.51, *p* = .18). For German sample, the variable age contributed 1.2% (*R*^2^ = .012). When statistically controlled for age, the change in *R*^2^ was 10.2% (*R*^2^ = .114). The model narrowly missed significance (*F* (6, 97) = 1.95, *p* = .08). The individual contribution of each predictor is given in Table 3.

**Table 3:** Stepwise regression analyses predicting HRQoL of Pakistani and German CHF patients, and results of Fisher’s z-tests, comparing *R*^2^ between the two samples

	Outcome	Predictors	Pakistan			Germany		
			*β*	*t*	*p*	*β*	*t*	*p*
Block 1	PCS	Age	.01	.12	.91	− .11	− 1.08	.28
Block 2		Age	.00	.001	.99	− .08	− .77	.44
		Trait EI	.05	.39	.69	.26	2.37	.02*
		DS	− 1.14	− 1.75	.08	.42	.76	.45
		QSS	.70	1.42	.16	.00	.001	1.00
		Trait EI x DS	.95	1.45	.15	− .47	− .84	.40
		Trait EI x QSS	− .61	− 1.26	.21	− .06	− .10	.92
Block 1	MCS	Age	.06	.55	.58	− .06	− .58	.56
Block 2		Age	− .003	− .03	.97	.001	.01	.99
		Trait EI	.42	3.87	.001***	.42	4.09	.001***
		DS	.64	1.07	.29	.15	.30	.76
		QSS	− .13	− .29	.77	− .41	− .75	.45
		Trait EI x DS	− .90	− 1.51	.13	− .25	− .49	.63
		Trait EI x QSS	.10	.24	.81	.26	.48	.63

*DS* discrepancy score in religious beliefs and practices, *MCS* mental component summary, *PCS* physical component summary, *QSS* quality of social support

**p ≤ .01, *** *p *≤ .001

In case of MCS, the analysis showed that for Pakistani sample, age contributed .3% (*R*^2^ = .003) while after controlling for age, the *R*^2^ changed 23% and the model was also significant (*F* (6, 98) = 4.65, *p* ≤ .001). Among the potential predictors trait, EI appeared to be the predictor of MCS among Pakistani CHF sample (*β* = .42, *t* = 3.87, *p *≤ .001). For German sample, after statistically controlling for the individual variable age (*R*^*2*^ = .004), the change in *R*^2^ was 23.8% and the model was significant (*F* (6, 97) = 4.82, *p* ≤ .001). In the German sample, trait EI was also the predictor of MCS among German CHF sample (*β* = .42, *t* = 4.09, *p *≤ .001).

It revealed that higher the trait EI in both the Pakistani and German CHF patients, better was their MCS of HRQoL when controlling for age and this effect was stronger in German CHF patients.

### Qualitative Difference Between Pakistan and Germany for Attributed Reasons of CHF

**Fig. 1:**
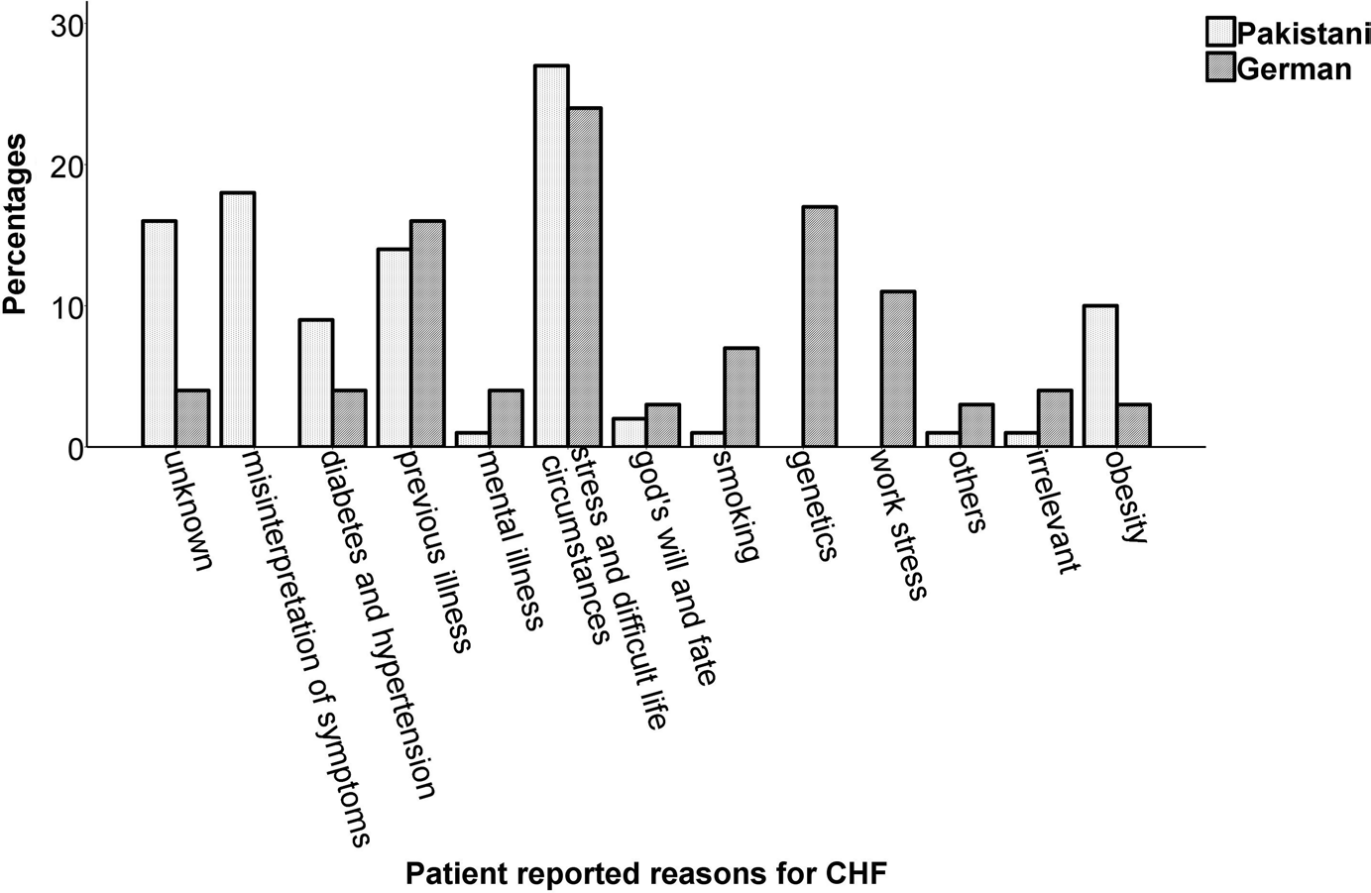
Percentage of different reasons of chronic heart failure (CHF) in the sample of (*N* = 100) Pakistani and German CHF individuals

The qualitative analyses revealed 13 prominent categories that the patients considered as a reason for their CHF (see Fig. 1). A considerable number of participants, in particular Pakistani, responded that they did not know the reason behind their illness (unknown). Category ‘others’ subsumes rarely mentioned factors such as age, medications, and excessive work. Overall comparison of these data suggested that the Pakistani CHF patients were significantly different from the German sample regarding awareness of the illness (*Chi*^2^ (68.50), *df *= 12, *ϕ* = .58, *p *< .001). However, post hoc analyses showed that there were no significant differences among individual categories between the two cultures (all *p*’s > .001).

### Measures Taken to Treat CHF

**Fig. 2:**
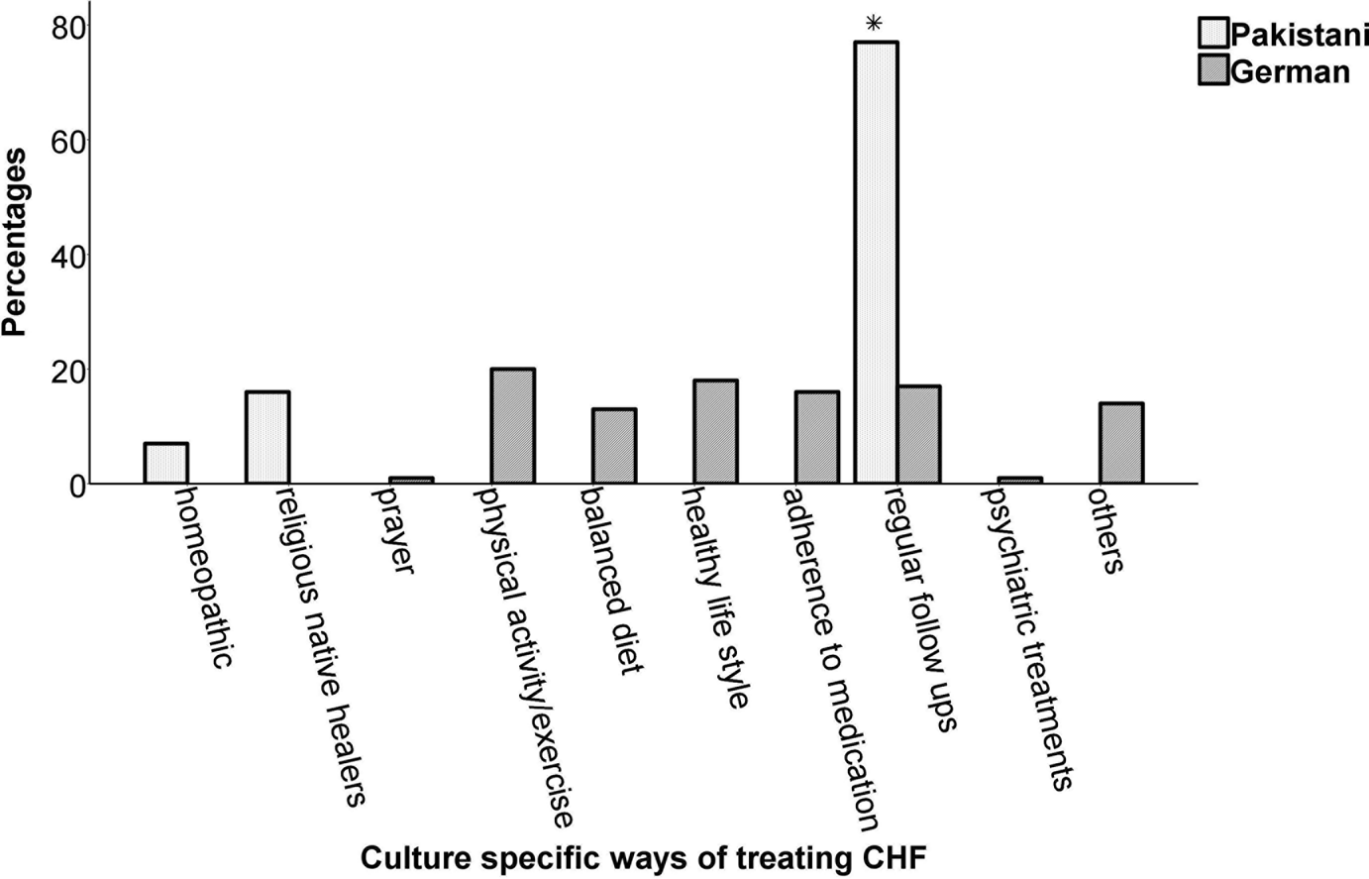
Percentage of different treatments for chronic heart failure (CHF) in the sample of (*N* = 100) Pakistani and German CHF individuals

The two cultures differed with regards to the type of treatment obtained (*Chi*^*2*^(144.29), *df* = 9, *ϕ* = .85, *p* < .001). Figure 2 shows that the Pakistani sample primarily sought treatment from religious native healers and homeopathic. In contrast, the German CHF patients showed a tendency to take precautionary measures to maintain healthy living such as maintaining healthy life styles (e.g., exercise, no smoking), adherence to medication and balanced diet. Post hoc analysis indicated that the two countries significantly differed in terms of follow-up visits to the hospital that were reported frequently by the Pakistani CHF patients (Pakistan *n* = 77, 77% vs. Germany *n* = 17, 17%, *p* < .0025).

### Presence of Common Worries in Life

**Fig. 3:**
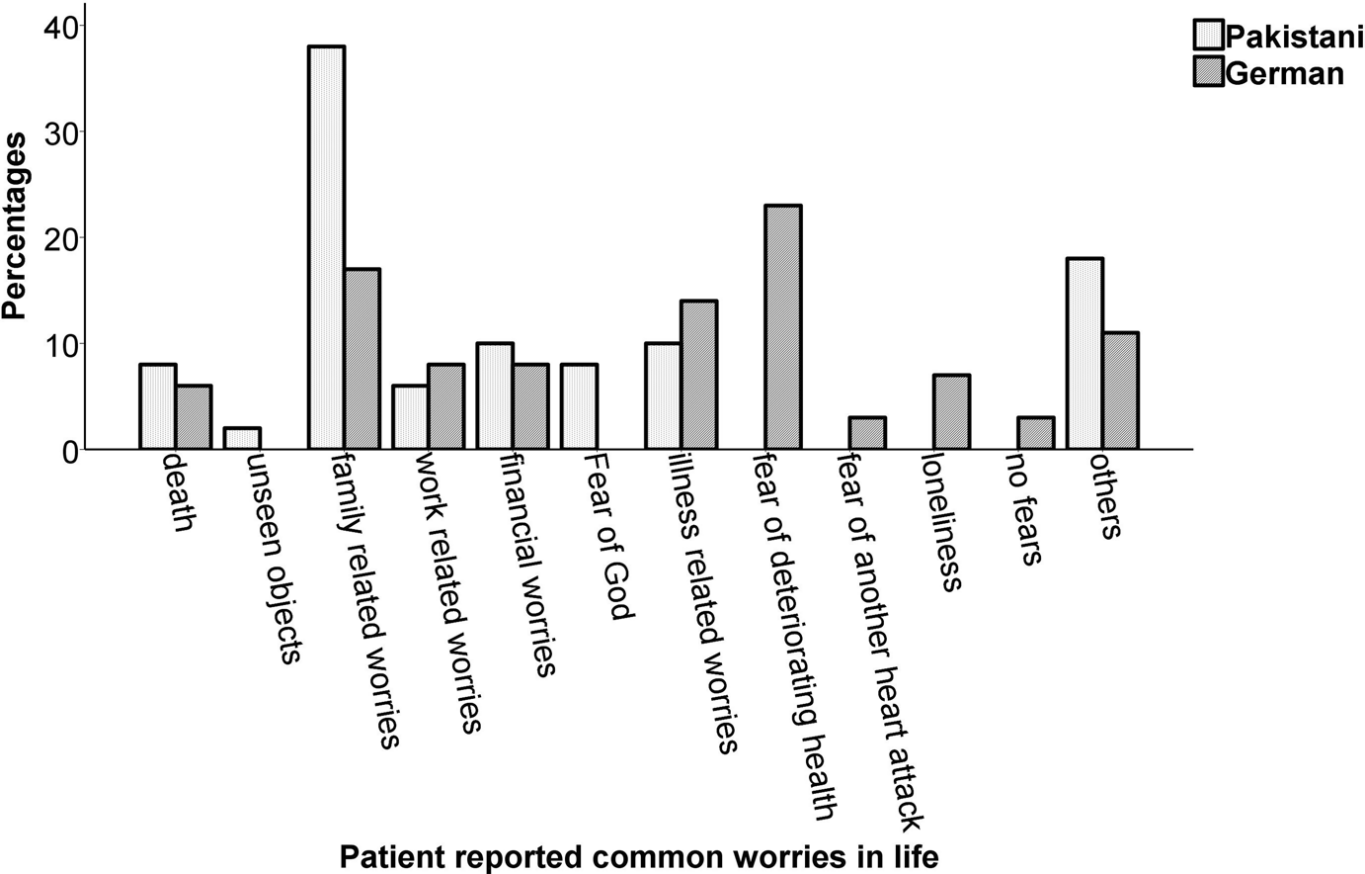
Percentage of common fears in the sample of (*N* = 100) Pakistani and German chronic heart failure (CHF) individuals

Participant’s responses were coded into 12 categories as evident from Fig. 3. Chi-square analysis indicated significant overall differences between Pakistan and Germany (*Chi*^*2*^(57.16), *df *= 11, *ϕ* = .53, *p* < .001). Family-related worries were the most common in both cultures. However, fear of deteriorating health was more prevalent in the German CHF patients as opposed to fear of God in the Pakistani sample. However, Post hoc analysis did not show any significant differences among individual categories between two cultures (*p* > .002).

### Measures Taken to Overcome Worries in Life

**Fig. 4:**
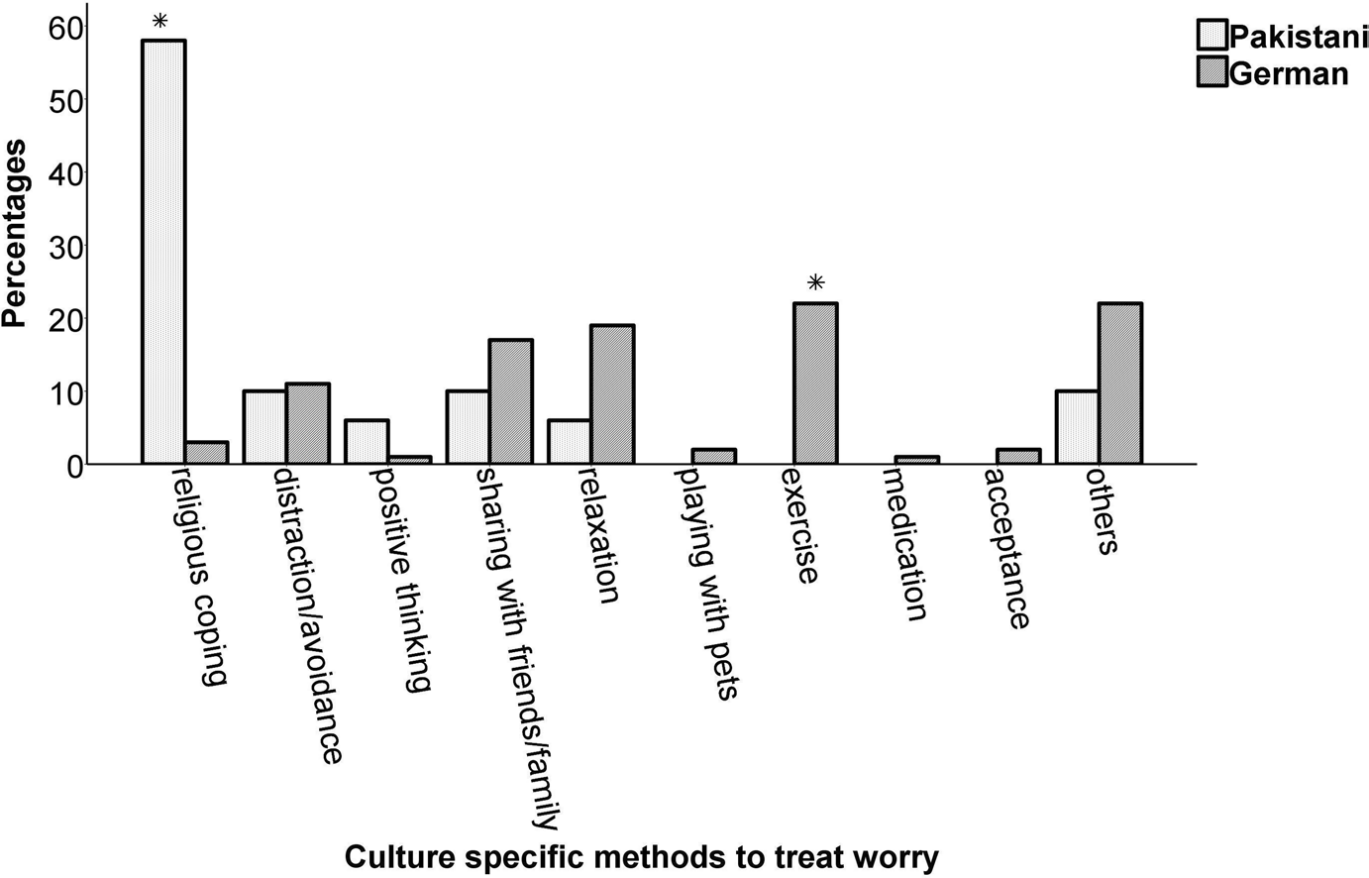
Percentage of different measures taken to overcome worry in the sample of (*N* = 100) Pakistani and German chronic heart failure (CHF) individuals

Figure 4 shows the ten categories revealed by analysis of measures to overcome worries in life. Chi square analysis indicated significant differences between the two cultures (*Chi*^2^ (93.28), *df *= 9, *ϕ* = .68, *p* < .001). Post hoc analysis showed that Germany and Pakistan differed significantly in terms of religious coping (Pakistan *n* = 58, 58% vs. Germany *n* = 3, 3%, *p* < .0025) and exercise (Germany *n* = 22, 22% vs. Pakistan *n* = 0, 0%, *p* < .0025).

## Discussion

The present study was a cross-cultural comparison between German and Pakistani cardiology patients on the roles of religion, social support and trait-EI as coping mechanisms in maintaining health-related quality of life (HRQoL). Additionally, the roles of trait-EI, discrepancy score in religious beliefs and practices (DS) as well as quality of social support (QSS) along with the interactions of trait EI with DS and QSS in predicting HRQoL were explored.

### Roles of Religion and Social Support Among Pakistani and German CHF Patients

The results revealed that despite similar levels of trait-EI, religion was more prevalent in the Pakistani sample. Yet, in the Pakistani CHF patients, the higher their trait-EI, the greater was the importance given to religion. This confirms earlier findings supporting the relationship between EI and religiosity (Butt 2014). However, the current findings did not support any significant relationship between trait-EI and self-rating of oneself as a religious practitioner in Pakistan, which is contrary to the results of Paek (2006). Interestingly, also the DS was higher in the Pakistani sample. This may be a source of distress, because the Pakistani CHF sample considered religion as very important, yet seemed to be low on how much they feel they have done of what they should do (i.e., religion). None of these relationships were found to be significant among the German CHF patients. This may be an expression of the mostly secular character of Germany.

Another important difference between cultures was that most of the Pakistani CHF patients lived in an extended family household as the main breadwinner and decision-maker. Part of the sample from Pakistani community also reported to be dependent on their children who were the primary financial supporter in a considerable number of families when controlled for marital status. Although not measured directly but it could be speculated from the given information that, first, this underlines the major impairment created by CHF. Second, these socioeconomical and family factors might have resulted in a failure to maintain a balance between the obligations owed to God and the responsibilities toward humanity and consequently in high DS for the patients themselves.

In the German sample, the patient alone or mutually with the spousal partner was the decision-maker. In addition, the majority of the German CHF patients lived in a nuclear family system. Interestingly, the German CHF patients comparatively reported better physical and mental component summaries of HRQoL. Perhaps living in a large family with limited financial resources in combination with physical and psychological health problems has fostered dysfunctional family coping after the onset of illness, financial burden, and less knowledge regarding illness in the Pakistani CHF patients. This may have affected the emotional processing of the Pakistani CHF patients and contributed to poor HRQoL. In contrast, the shared responsibility in German spousal relationships may have contributed to more healthy social roles and interactions that focus on finding an adequate level of support. In large family systems, overprotective social support may be more common. According to the self-determination theory (Ryan and Deci 2000) and the stress buffering social support hypothesis, this could even be a factor promoting the progression of CHF (Zniva et al. 2016). Future studies should therefore examine the cultural difference regarding the relationship between family systems, type of social support and underlying factors as for example suggested by the self-determination theory (Ryan and Deci 2000).

This view is further in line with the evaluation of social support in the two cultures. First, overall social support appeared to be a hallmark of German society. Although no cultural differences were observed regarding the importance assigned to their families, the German CHF patients assigned more importance to friends as a social support group. This strengthens the argument, that for the Pakistani patients, problems may arise due to limited resources and the associated negative consequences of ill-adjusted coping within their family systems, warranting the attention of health practitioners toward this issue.

### Trait-EI and HRQoL

Higher trait-EI predicted better mental component summary of HRQoL in both German and Pakistani CHF patients, after controlling for age. However, this effect was stronger in German CHF patients. Since absolute levels of trait-EI were similar in both countries and the age was statistically controlled, other characteristics are likely to explain this slightly stronger effect in German CHF patients. It is evident from the current qualitative data that the German CHF patients have a high internal locus of control, reflected in their high consciousness regarding healthy living styles and a positive attitude regarding exercise as well as mental and physical component summaries of HRQoL. In contrast, the Pakistani CHF patients showed an external locus of control reflected by the importance they assigned to religion and putting their trust and the associated control into the hand of “God” (Krause 2002). Locus of control theory (Rotter 1966) suggests that the Pakistani patients therefore took a less active role in dealing with stress, which has been confirmed by the current findings (i.e., poor mental and physical component summaries of HRQoL, less exercise, and more religious coping in an effort to overcome worries in life). In line with this view, the results also indicated that the Pakistani CHF patients sought external help (medical, religious native healers, homeopathy, and/or religion itself) to cope with CHF.

Another interesting finding was that the Pakistani patients worried more about their family (“what will happen to them if I die”) than their own health. This attitude fits a collectivistic ideology that is obviously more prominent in Pakistan than Germany. Unfortunately, this may contribute to a more interdependent self-image, or in other words stronger external locus of control, which may undermine experiences of autonomy and self-efficacy.

### Limitations and Future Directions

Although these may be important and theoretically well-supported aspects that could explain the cultural differences found in the current study, other characteristics may also contribute. For example, considering Germany one of the leading industrialized nations, while Pakistan is counted under the developing countries, future research should focus on factors such as feasible access of professional support (appointment, medicine). In addition, age was found to be significantly different between the two samples making the German CHF sample approximately eleven years older yet with better HRQoL than the Pakistani CHF sample. Future research should focus on the socio-economic factors and their effect on age in different societies such as Pakistan and Germany. Moreover, provision and implementation of modern web-based intervention can help improve psychosocial well-being of CHF individuals. Recently a multi-centered six-week web-based randomized controlled trial was found significantly effective in alleviating anxiety and depression and improving psychosocial well-being of heart failure patients. The patients showed significant improvement (compared to the control group) at six weeks and one-year post-assessment (Schulz et al. 2019). Similar trials focusing factors responsible for escalating psychosocial burden in CHF patients should be implemented cross-culturally.

Albeit we aimed for parsimonious, yet plausible and ecologically valid explanations, all causal interpretations need to be made with caution. Future research with prospective and experimental designs will show whether the hypotheses generated from these approaches can be confirmed. In these studies also the situation-specific importance of religion, in particular concerning how religion and the importance associated with it contributes to each person’s life individually, may be an important issue. In this respect, it is suggested to replicate the study, however with not only a dimensional but temporal focus on religion and its associated adaptive outcomes in CHF individuals. Finally, it should be noted that the current findings cannot be generalized to all socioeconomic classes as well as all Muslims and Christian communities across the globe, since religious believe and practice greatly varies along with nationalities and associated cultures.

## Conclusion

The current findings highlight the role of higher trait-EI for better mental component of HRQoL in the Germany and Pakistani CHF patients. The effect was stronger in German CHF patients. In the Pakistani CHF patients, external locus of control may explain the lack of the positive effect of high trait-EI on HRQoL. These findings show options for culture-specific tailoring of therapeutic and social support that may aim for strengthening self-efficacy and lowering the distress created by high levels of DS in the Pakistani sample. In the German CHF patients, resource-oriented approaches may further strengthen the positive effects of problem-solving-oriented strategies such as implementing a healthy life style and may further optimize the utility of social support. Future studies may confirm the effectiveness of such approaches in experimental and prospective designs.
